# Improving the competence and confidence of pulmonary and critical care medicine fellows in performing a cricothyrotomy

**Published:** 2019-07-24

**Authors:** Kapil Rajwani, Elizabeth Mauer, Timothy Clapper

**Affiliations:** 1 Department of Medicine, Weill Cornell Medical College, New York, USA; 2 Department of Healthcare Policy & Research, Weill Cornell Medical College, New York, USA; 3 Weill Cornell Medicine New York-Presbyterian Simulation Program & Center, Weill Cornell Medical College, New York, USA

## Abstract

**Implication Statement**

Clinical opportunities to practice or perform a cricothyrotomy are limited. We developed an evidence-based cricothyrotomy course following the 4-phase lesson plan for simulation that provides pulmonary and critical care medicine fellows with demonstrations, practice, and feedback to increase their confidence and competence. Survey results demonstrated an improvement in perceived confidence (p<0.005) and competence (p<0.002) following this educational intervention. Fellows also achieved significant improvement in knowledge (p<0.003) and performance in two cricothyrotomy techniques (Seldinger and MacIntyre) (p<0.004). It is important that we provide fellows with practice opportunities that can be used to develop and maintain proficiency, particularly in low frequency events.

## Introduction

The cricothyrotomy is a rare procedure resulting in few clinical opportunities to apply it. Practice opportunities are also limited because of the constrained time that pulmonary and critical care medicine fellows have available to education, along with competing requirements to maintain proficiency in other skills. In our organization, the cohort of pulmonary critical care fellows (n=11), years 1-3 requested focused simulation training to address potential deficits.

In this article, we describe the innovative method of instruction that we used to improve pulmonary and critical care medicine fellow knowledge and skills when performing cricothyrotomies.

## Intervention

Participants provided informed consent to this Institutional Review Board approved research prior to the intervention. To maximize participation in this education intervention and minimize clinical interruption, we divided the 3-hour course into two hour and one-half sessions using the four-phase lesson plan for simulation.^1-2^ During the *Inquire phase*, the fellows reflected on their past use and knowledge of cricothyrotomies. In the *gather phase*, the fellows were provided with topic sheets that summarized current research findings from the literature. We also provided the fellows with a demonstration and practice locating cricothyrotomy landmarks with and without the use of ultrasound^3^ on live male and female volunteers. The fellows then practiced multiple times over a 3-D Model and a porcine model trachea using the Seldinger technique and MacIntyre-3-step bougie assisted technique.^4^ Returning exactly two weeks later, the fellows participated in the *process phase*, which included high-fidelity simulations involving difficult airway cases and the need to perform a cricothyrotomy. We debriefed the fellows in the *apply phase* and asked them to reflect on their performance and ways that they would apply the skills to their clinical setting.

We surveyed the fellows’ past use of cricothyrotomies, current and post-intervention comfort, and evaluated both methods *using the instructions* from the cricothyrotomy Seldinger kit (Cook Medical^5^) and article by MacIntyre.^4^ We added identical pre and post-procedural steps to both instruments including, gathering and prepping equipment, patient positioning, identifying landmarks and confirming correct tube placement, suctioning, and device securement. They received one point if the step was performed properly. None of the fellows had placed a cricothyrotomy on an actual patient prior to our training.

## Results

We analyzed changes in knowledge and performance by obtaining p-values from Wilcoxon signed-rank tests. Survey data were calculated by percentage and recurring themes. As shown in Table 1, our fellows improved in all areas. Overwhelmingly, the fellows reported that they were more likely to perform a cricothyrotomy as a result of the training. With limited research surrounding how long training benefits last, some next steps would be to investigate retention over designated periods. Limitations of our research include the small sample size of our pilot study at one site. Multi-site research with a larger sample would be useful, as well as results that demonstrate significance at the patient level.

**Table 1:** Survey, knowledge, and procedural score results

	N	Mean	SD	Median	Min	Max	P
**Perceived competence (rating 1-4 with 4 being highest)**							
Pre	11	1.91	0.54	2	1	3	0.002
Post	11	3.18	0.6	3	2	4	
**Perceived confidence (rating 1-4 with 4 being highest)**							
Pre	11	1.64	0.81	2	0	3	0.005
Post	11	3.09	0.7	3	2	4	
**Knowledge test results pre and post intervention (10-question test assessing knowledge of cricothyrotomies)**							
Pre	11	6.09	0.94	6	5	8	0.003
Post	11	7.73	0.9	8	6	9	
**Cricothyrotomy procedure results (20-point checklist with one point per procedural step)**							
**Seldinger Technique**	N	Mean	SD	Median	Min	Max	P
Pre	11	13.82	3.71	15	5	18	0.004
Post	11	19.45	0.52	19	19	20	
**MacIntyre Technique**	N	Mean	SD	Median	Min	Max	P
Pre	11	12.09	3.62	14	5	15	0.004
Post	11	16.64	0.5	17	16	17	

## Conclusion

The Accreditation Council for Graduate Medical Education lists airway management as a competency that pulmonary-critical care fellows must achieve during their fellowship training. Front of neck access is an essential part of the difficult airway algorithm. Since this situation remains relatively rare, the opportunities to practice are limited. In our pilot study, we demonstrated that a learner-requested, simulation intervention can improve pulmonary critical care fellow confidence and competence in placement of a cricothyrotomy. All three levels of fellowship benefited from this educational intervention and the fellows greatly appreciated the timely faculty response to their request for training. Of course, application of the skills is very important. During a recent case, one of the fellows performed a cricothyrotomy on a patient and was commended by senior faculty for her calmness and skill while saving the patient’s life. With an increasing amount of critical skillsets, it is important that we provide fellows with knowledge and practice opportunities that can be used to develop and maintain proficiency, particularly in low frequency events. We encourage faculty to try this intervention with their fellows.
